# Cell Envelope Integrity and Capsule Characterization of Rhodotorula mucilaginosa Strains from Clinical and Environmental Sources

**DOI:** 10.1128/mSphere.00166-19

**Published:** 2019-06-05

**Authors:** Johnathan Yockey, Luke Andres, Moleigh Carson, Jeramia J. Ory, Amy J. Reese

**Affiliations:** a St. Louis College of Pharmacy, St. Louis, Missouri, USA; Carnegie Mellon University

**Keywords:** *Cryptococcus*, *Rhodotorula*, capsule, cell wall integrity

## Abstract

*Rhodotorula* yeasts are pink, encapsulated basidiomycetes isolated from a variety of environments and clinical settings. They are increasingly linked with disease, particularly central venous catheter infections and meningitis, in immunocompromised patients. Eight clinical and eight environmental strains molecularly typed as Rhodotorula mucilaginosa were compared to six Cryptococcus neoformans strains for phenotypic variability. Growth on cell integrity-challenging media suggested that R. mucilaginosa cells possess differences in signaling pathways, cell wall composition, or assembly and that their membranes are more susceptible to perturbations than those of C. neoformans. All 16 R. mucilaginosa strains produced urease, while none produced melanin with l-3,4-dihydroxyphenylalanine (l-DOPA) as a substrate. India ink staining reveals that clinical R. mucilaginosa capsules are larger than environmental capsules but that both are generally smaller than C. neoformans capsules. All R. mucilaginosa strains were resistant to fluconazole. Only two clinical strains were susceptible to voriconazole; all of the environmental strains were resistant. We generated an anticapsular antibody (Rh1) to R. mucilaginosa; Rh1 did not bind C. neoformans control strains, was specific to *Rhodotorula* species, and bound to all tested *Rhodotorula* strains. Binding assays performed with wheat germ agglutinin (WGA), concanavalin A (ConA), calcofluor white (CFW), and eosin Y dye (EY) cell surface probes suggested that chitin may be more accessible in R. mucilaginosa but that the total abundance of chitooligomers is less than in C. neoformans. This report describes a novel reagent that can be used to identify *Rhodotorula* species and lays the foundation for future cell envelope composition analysis.

**IMPORTANCE** Currently, there is very little known about the phenotypic variability within species of *Rhodotorula* strains and the role of their capsule. Cryptococcus neoformans has been considered the only encapsulated human fungal pathogen, but as more individuals come to live in states of immunocompromised health, they are more susceptible to fungal infections, including those by *Rhodotorula*. R. mucilaginosa species are some of those most commonly associated with clinical infections. We wanted to know if clinical and environmental strains of R. mucilaginosa demonstrated disparate capsule phenotypes. With limited antifungal options available and clinical *Rhodotorula* spp. often resistant to common antifungal drugs such as fluconazole, caspofungin (1, 2), and voriconazole (2), a better understanding of the fungal biology could inform the design and use of future antifungal drugs. The generation of an antibody specific to *Rhodotorula* fungi could be a useful diagnostic tool, and this work presents the first mention of such in the literature.

## Abstract (precis)

Currently, there is very little known about the phenotypic variability within species of *Rhodotorula* strains and the role of their capsule. Cryptococcus neoformans has been considered the only encapsulated human fungal pathogen, but as more individuals come to live in states of immunocompromised health, they are more susceptible to fungal infections, including those by *Rhodotorula*. R. mucilaginosa species are some of those most commonly associated with clinical infections. We wanted to know if clinical and environmental strains of R. mucilaginosa demonstrated disparate capsule phenotypes. With limited antifungal options available and clinical *Rhodotorula* spp. often resistant to common antifungal drugs such as fluconazole, caspofungin (1, 2), and voriconazole (2), a better understanding of the fungal biology could inform the design and use of future antifungal drugs. The generation of an antibody specific to *Rhodotorula* fungi could be a useful diagnostic tool, and this work presents the first mention of such in the literature.

## INTRODUCTION

With advances in medicine, people are living longer in immunosuppressed states, making them more susceptible to fungal infections of various types that range from mild to severe. The literature demonstrates an increasing number of reports of *Rhodotorula* spp. being isolated from patients, with R. mucilaginosa being the most common species. Central venous catheter (CVC) usage has been linked most extensively with fungemia in immunocompromised patients (3–6), while human immunodeficiency virus (HIV)-positive status has been linked most extensively with cases of meningitis (2, 4).

One particular challenge with *Rhodotorula* infections is efficient identification of the etiologic agent, as *Rhodotorula* species are not in the short list of those likely to cause fungal infections. A second concern is the practice of treating fungal infections with echinocandins, such as caspofungin, before complete identification has been made. This class of antifungal drugs is not effective against *Rhodotorula* and other basidiomycetes, such as Cryptococcus neoformans (7). Susceptibility tests have demonstrated that *Rhodotorula* species respond best to amphotericin B and flucytosine and poorly to the azoles (1, 7–10), with amphotericin B still being the primary drug of choice. Such susceptibility methods have not been employed with environmental strains.

The cells of *Rhodotorula* spp. are generally oval-shaped cells that yield pink to coral-colored colonies on standard yeast media (11). Cell shapes do seem to differ between and within *Rhodotorula* spp., with some being considerably more rod-like (12–14). Cell wall composition may play a role in these shape differences, as has been shown in other fungi and reviewed previously by Bose et al. (15). The wall composition and stability of C. neoformans cells have been studied by various laboratories using media containing stress-inducing components such as Congo red, salt, calcofluor white, sodium dodecyl sulfate, caffeine, and hydrogen peroxide (16–18), but these types of cell integrity-challenging phenotypic assays have not been widely explored for *Rhodotorula* strains.

*Rhodotorula* cells have been reported to exhibit a thin layer of capsule (11). While the capsule of C. neoformans has been the focus of many studies, including those performed with India ink and fluorophore-tagged anticapsule antibodies, very little is known about the surface or capsule of *Rhodotorula* species. To date, there has been one report of binding of concanavalin A to environmental *Rhodotorula* strains (Rhodosporidium toruloides) (14); no other studies of surface-binding probes, reports of capsule-binding antibodies, or India ink images of capsule have been published for *Rhodotorula* strains.

For this study, we were interested in whether or not R. mucilaginosa strains isolated from patients and the environment had particular phenotypic profiles and how these strains differed from those of C. neoformans. Only one study to date compared various species of environmental and clinical *Rhodotorula* strains (10), and the focus was on biofilm formation. Comparisons between *Rhodotorula* and *Cryptococcus* species appear only in passing in the literature. For the purpose of this work, we focused on cell wall integrity studies, the production of virulence factors of melanization and urease, antifungal disk diffusion susceptibility, capsule characterization, and cell surface analysis by fluorescent probes to expand our understanding of R. mucilaginosa variability and to compare this emerging pathogen to the well-studied species C. neoformans.

## RESULTS

### Only strains that were genotyped as R. mucilaginosa were selected for the study.

**FIG 1:**
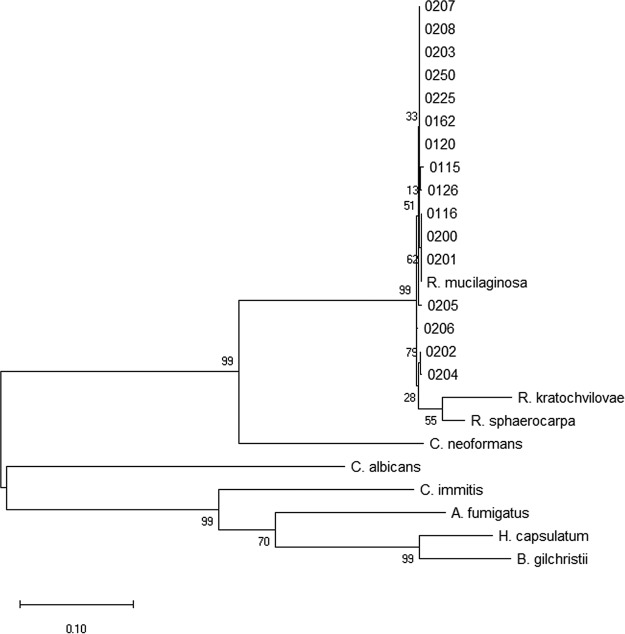
Phylogenetic analysis of the ITS regions shows that the 16 R. mucilaginosa strains form a clade separate from other *Rhodotorula* spp. and from other basidiomycetes. The evolutionary history was inferred by using the maximum likelihood method based on the Kimura 2-parameter model (37). The tree with the highest log likelihood value (−4,170.28) is shown. The tree is drawn to scale, with branch lengths measured in the number of substitutions per site. The analysis involved 25 nucleotide sequences. All positions with less than 95% site coverage were eliminated. There were a total of 785 positions in the final data set. Evolutionary analyses were conducted in MEGA7 (38). *R. kratochvilovae*, *Rhodotorula kratochvilovae*; R. sphaerocarpa, Rhodotorula sphaerocarpa; A. fumigatus, Aspergillus fumigatus; H. capsulatum, Histoplasma capsulatum; B. gilchristii, Blastomyces gilchristii.

All putative *Rhodotorula* strains of interest yielded a DNA amplification product in the expected range of 500 bp using ITS1 and ITS4 primers and were successfully subcloned into TOPO TA vectors. These clones were successfully sequenced, trace data were assembled, and the regions were compared to database sequences. This allowed us to select and proceed with only those strains that matched R. mucilaginosa for further comparative analysis (see Table 1) (Fig. 1), including all eight of our clinical strains and a subset of our environmental strains.

**TABLE 1:** *Cryptococcus* and *Rhodotorula* strains utilized throughout experimental methods^*a*^

ARY strain	Sample origin	Sample category
*Cryptococcus*		
177	NIH 117	Control, clinical
178	NIH 192	Control, clinical
179	NIH 430	Control, clinical
180	NIH 433	Control, clinical
101	H99, clinical strain (var. *grubii*, serotype A)	Control, clinical
105	JEC21, clinical strain (var. *neoformans*, serotype D)	Control, clinical

*Rhodotorula*		
115	St. Louis, MO; Washington University; laboratory contaminant; 2002	Environmental
116	Allentown, PA; Cedar Crest College; campus isolate; 2004	Environmental
120	Allentown, PA; Cedar Crest College; laboratory contaminant	Environmental
126	Allentown, PA; Grace Montessori School	Environmental
162	Cumberland, PA; avian dropping swab	Environmental
200	Ward’s no. 470179-708 plate or Carolina no. 156260 slant; listed as R. rubra	Environmental (purchased)
201	UICCOM; blood sample from Madrid, Spain; 2001	Clinical
202	UICCOM; blood sample from Johannesburg, South Africa; 2008	Clinical
203	UICCOM; blood sample from Hershey, PA; 2007	Clinical
204	UICCOM; blood sample from Johannesburg, South Africa; 2004	Clinical
205	UICCOM; blood sample from Szeged, Hungary; 2007	Clinical
206	UICCOM; blood sample from Bratislava, Slovakia; 2001	Clinical
207	UICCOM; blood sample from Edmonton, Canada; 2003	Clinical
208	UICCOM; blood sample from Johannesburg, South Africa; 2007	Clinical
225	Allentown, PA; Cedar Crest College; tree sample	Environmental
250	Amberson, PA; golden retriever dog coat sample	Environmental

a The number indicates strains from the Amy Reese yeast (ARY) collection. The purchased strain is described as R. rubra but is cross-listed with ATCC 9449, NRRL Y-1592, and MUCL 30397, which are listed as R. mucilaginosa (Jorgensen) F.C. Harrison (1928). Strains from blood samples were obtained from the University of Iowa Carver College of Medicine (UICCOM) (1).

### R. mucilaginosa strains were less tolerant than C. neoformans strains to Congo red and more tolerant to caffeine on YPD plate media.

**FIG 2:**
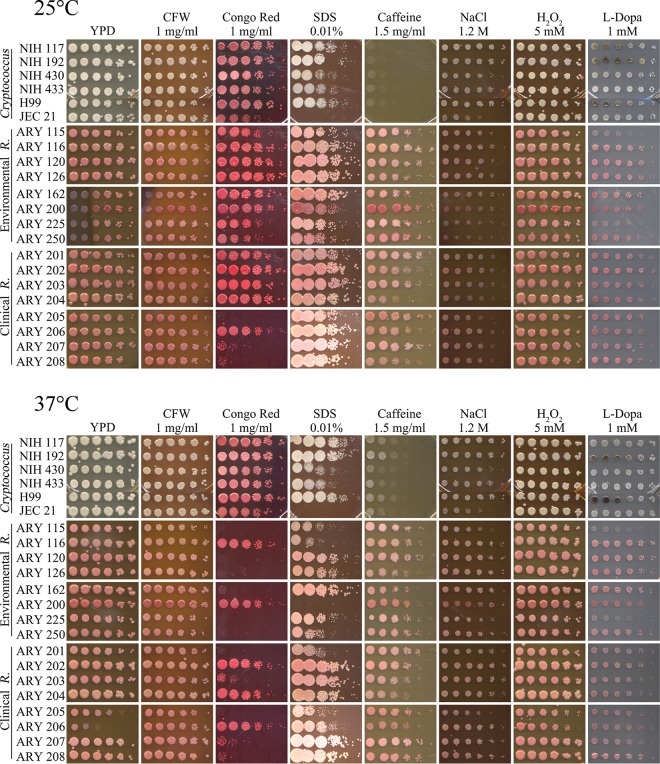
R. mucilaginosa strains have cell wall polymer compositions that differ from those of C. neoformans strains and do not exhibit melanization. Serial dilutions of six strains of C. neoformans (NIH 117 to 433 [as indicated], H99, and JEC21), eight environmental strains of R. mucilaginosa, var. *neoformans* (ARY 115 to 250 [as indicated]), and eight clinical strains of R. mucilaginosa (ARY 201 to 208 [as indicated]) were spotted onto YPD agar media containing cell envelope integrity-challenging ingredients or on minimal media with l-DOPA as indicated. The initial spot was 1 × 10^6^ cells/ml, and subsequent spots were 5-fold dilutions. R. mucilaginosa strains were more susceptible to 1 mg/ml Congo red and 0.01% SDS than C. neoformans cells but were more robust in the presence of 1.5 mg/ml caffeine in YPD agar media. R. mucilaginosa strains did not melaninize. (Top) Plates were incubated at 25°C for 4 days. (Bottom) Plates were incubated at 37°C for 4 days.

We employed serial dilution plating of our R. mucilaginosa and C. neoformans strains under various cell wall integrity conditions to access their behavior. All of our strains grew well at 25 and 37°C on plain yeast extract-peptone-dextrose (YPD) agar media, although growth of strain ARY 206 (Amy Reese yeast [ARY] collection) appeared to be slightly less robust on YPD agar alone at 37°C in the shown trial than in some of our previous tests, where it grew in a manner that showed greater similarity to the growth seen with other strains. We therefore concluded that the growth changes seen on various media were related to the change in conditions and not to changes in temperature (Fig. 2).

Calcofluor white (CFW; also referred to as fluorescent brightener) disrupts the normal assembly of chitin microfibrils in the cell wall (17, 19). R. mucilaginosa strains plated on YPD media containing 1 mg/ml CFW were similar to the tested C. neoformans strains in growth characteristics (Fig. 2). Our data suggest a similar lack of impact of CFW on chitin formation in the cell walls in comparisons between the two fungi.

Congo red has been demonstrated to bind to fungal cell wall beta-d-glucans (20, 21) and/or to chitin (19, 21), interfering with cell wall construction. While the cryptococcal strains all grew robustly with 1 mg/ml Congo red in the YPD agar media at both 25 and 37°C, *Rhodotorula* strains were more profoundly inhibited, especially at 37°C (Fig. 2). Only two of the environmental strains showed any notable growth, while three of the clinical strains showed notable growth at 37°C. This suggests differences in the cell wall polymers of R. mucilaginosa strains in comparison to C. neoformans strains, likely in the cell wall beta-d-glucans and/or chitin composition or their assembly.

Sodium dodecyl sulfate (SDS) interrupts cell membranes and may cause cells with less-robust membranes to lyse (16, 22). The cryptococcal strains grew to the same order of magnitude in YPD agar with 0.01% SDS at both 25 and 37°C, with the notable exception being the JEC 21 strain, which failed to thrive (and which is a different serotype). The same was true for R. mucilaginosa, except for the purchased ARY 200 R. mucilaginosa strain, which did not grow at all at 37°C (Fig. 2). ARY 115, 116, 250, 201, and 205 did not exhibit growth to the same order of magnitude at 37°C (Fig. 2b). We tested all strains on media with 0.01% SDS because the R. mucilaginosa strains exhibited zero growth at either temperature when we initially tested media with 0.05% SDS. This result led us to believe that the the characteristics of membrane construction, stability, or accessibility in R. mucilaginosa strains may be different, as the rhodotorular cells were more susceptible to perturbations.

As a purine analog, caffeine can interfere with signal transduction pathways involved in cell wall production (23). The results seen with YPD agar with 1.5 mg/ml caffeine plating (Fig. 2) suggest that R. mucilaginosa strains are more robust in their growth than C. neoformans in the presence of this level of caffeine. Lighter growth of the cryptococcal strains has also been observed in the published spot-plating images for C. neoformans strains on 1 mg/ml caffeine (24). All of the R. mucilaginosa strains, both environmental and clinical, grew more robustly than cryptococcal strains at both 25 and 37°C (Fig. 2). This suggests differences in the signal transduction pathways between the two fungi and that R. mucilaginosa cells are compensating in cell structure integrity.

Sodium chloride has been utilized in plate media to test fungal cell wall osmotic stability (17, 19, 24). Hydrogen peroxide addition to media has been used to evaluate cell tolerance of oxidative stress (25, 26). YPD agar plates containing either 1.2 M NaCl or H_2_O_2_ did not appear to impact R. mucilaginosa cells differently from C. neoformans cells (Fig. 2). The two fungal species seem equally tolerant to osmotic and oxidative stresses under these conditions.

### Environmental and clinical R. mucilaginosa strains produce urease.

Production of urease is a virulence factor in C. neoformans (27). We were interested in determining whether all of the clinical and environmental strains would demonstrate production of urease and whether the strains would hydrolyze urea at similar rates. All 16 R. mucilaginosa strains and both tested C. neoformans strains, H99 and NIH 117, were positive for urease production in urea broth after 7 days at 37°C, while uninoculated media did not change color. Monitored for light or bright pink color, a larger but insignificant (*P* = 0.19 by Student's *t* test) number of the environmental R. mucilaginosa strains turned pink in the first few days (average of 3.5 days) than of the clinical strains (average of 4.5 days) or the C. neoformans strains (average of 5 days).

### R. mucilaginosa strains do not produce melanin.

The activity of laccase in producing melanin from substrates plays a role in cryptococcal virulence (28). Medium containing minimal glucose and l-3,4-dihydroxyphenylalanine (l-DOPA) has been demonstrated to induce the production of melanin in cells able to perform melanization (18). This process can be visualized by observing a blackening of the cells grown on the plate. The results seen with the 1 mM l-DOPA plates (Fig. 2) demonstrated consistent and pronounced melanization at 25 and 37°C in three C. neoformans strains (NIH 117, NIH 192, and H99), while no impact on any of the 16 R. mucilaginosa strains was seen at either 25 or 37°C.

### R. mucilaginosa strains are less susceptible to fluconazole and voriconazole than C. neoformans strains.

Clinical strains of R. mucilaginosa have been analyzed for their susceptibility to disks of fluconazole and voriconazole (1), and we were motivated by curiosity to employ the same methods for comparisons of our environmental strains to the clinical strains. The clinical strains were likely investigated as a part of a study reported previously by Pfaller et al. (1), but we did not have those data for our strains of interest, so we evaluated them in this study, comparing them to our environmental strains and four cryptococcal controls. Zones were photographed and measured from plates incubated at 37°C for 2 days. C. neoformans zones of inhibition for all four strains tested showed susceptibility to voriconazole (i.e., the zones were 17 mm in diameter) (1). Two of the C. neoformans strains were susceptible (S) to fluconazole, and two were susceptible in a dose-dependent manner (susceptible dose dependent [SDD]) (S = 19 mm in diameter, SDD = 15 to 18 mm) (1) (Table 2). None of the R. mucilaginosa strains yielded any zones of inhibition in response to fluconazole. Environmental R. mucilaginosa strains 120 and 162 produced zones of inhibition in response to voriconazole (measured at 10 mm each in diameter), but since the zones were less than 14 mm in diameter, they therefore would be considered to represent resistance (1). Clinical R. mucilaginosa strains 202 and 204 showed zones of inhibition that were 23 mm and 29 mm in diameter, indicating that both were susceptible to voriconazole (Table 2).

**TABLE 2:** Two clinical R. mucilaginosa strains were susceptible to voriconazole, while no environmental strains were susceptible to voriconazole and all R. mucilaginosa strains were resistant to fluconazole^*a*^

Strain	Zone of inhibition (mm), susceptibility rating	
	Voriconazole	Fluconazole
*Cryptococcus*		
H99	28, S	18, SDD
NIH 117	29, S	20, S
NIH 192	34, S	18, SDD
NIH 430	37, S	32, S

*Rhodotorula*, environmental		
ARY 115	0, R	0, R
ARY 116	0, R	0, R
ARY 120	10, R	0, R
ARY 126	0, R	0, R
ARY 162	10, R	0, R
ARY 200	0, R	0, R
ARY 225	0, R	0, R
ARY 250	0, R	0, R

*Rhodotorula*, clinical		
ARY 201	0, R	0, R
ARY 202	23, S	0, R
ARY 203	0, R	0, R
ARY 204	29, S	0, R
ARY 205	0, R	0, R
ARY 206	0, R	0, R
ARY 207	0, R	0, R
ARY 208	0, R	0, R

a C. neoformans (H99, NIH 117 to 430 as indicated), R. mucilaginosa environmental (ARY 115 to 250 as indicated), and R. mucilaginosa clinical (ARY 201 to 208 as indicated) strains were grown in YPD medium overnight at 30°C, and equivalent cell amounts were plated onto Mueller-Hinton plates. Two disks for each antifungal agent were placed onto each plate, and the plates were incubated for 2 days at 37°C before imaging and measuring. Zone-of-inhibition measurements (in millimeters) are listed followed by the susceptibility rating of susceptible (S), susceptible dose dependent (SDD), or resistant (R) as defined previously by Pfaller et al. (1). All of the C. neoformans strains were susceptible to voriconazole (zone of inhibition, over 17 mm [1]). Two environmental R. mucilaginosa exhibited zones of inhibition in response to voriconazole, but with zones of inhibition of less than 14 mm, they are considered resistant (1). Two clinical R. mucilaginosa strains were susceptible to voriconazole. Two of the C. neoformans strains were susceptible to fluconazole, and two were susceptible dose dependent (S = 19 mm; SDD = 15 to 18 mm [1]). None of the R. mucilaginosa strains yielded any zones of inhibition in response to fluconazole.

### R. mucilaginosa strains do not make capsules as large as those made by C. neoformans strains under standard conditions.

**FIG 3:**
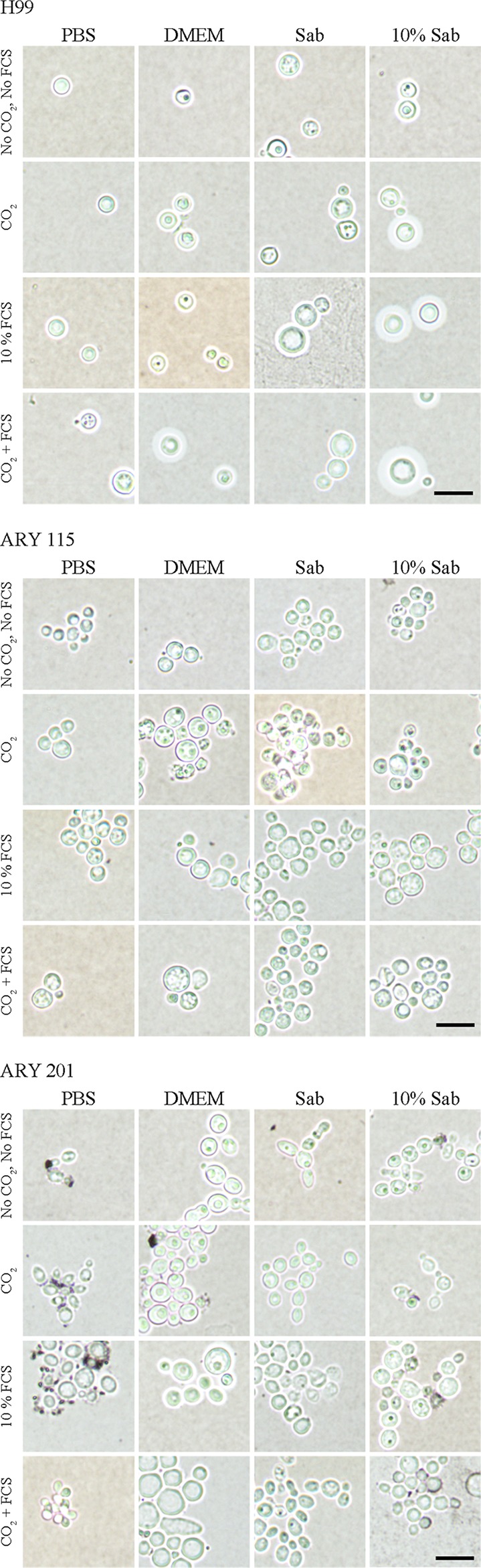
R. mucilaginosa strains do not produce capsules as large as those of C. neoformans under standard capsule-inducing conditions. Sixteen growth conditions are shown for the following three example strains: H99 C. neoformans, ARY 115 environmental R. mucilaginosa, and ARY 201 clinical R. mucilaginosa. Equivalent numbers of cells were introduced to wells containing PBS, DMEM, Sabouraud (Sab), or 10% diluted Sabouraud media (as indicated), with or without CO_2_ and with or without 10% FCS, and incubated for 1 week at 37°C. The experiments performed under the indicated CO_2_ conditions were performed at the same time for all strains. Samples of each well were mixed with India ink and visualized. While the DMEM and 10% Sabouraud conditions appeared to produce the largest capsules for H99, the R. mucilaginosa capsules were not nearly as large under any of the conditions. Bar, 10 μM. All images were taken at the same resolution and prepared in the same manner.

**FIG 4:**
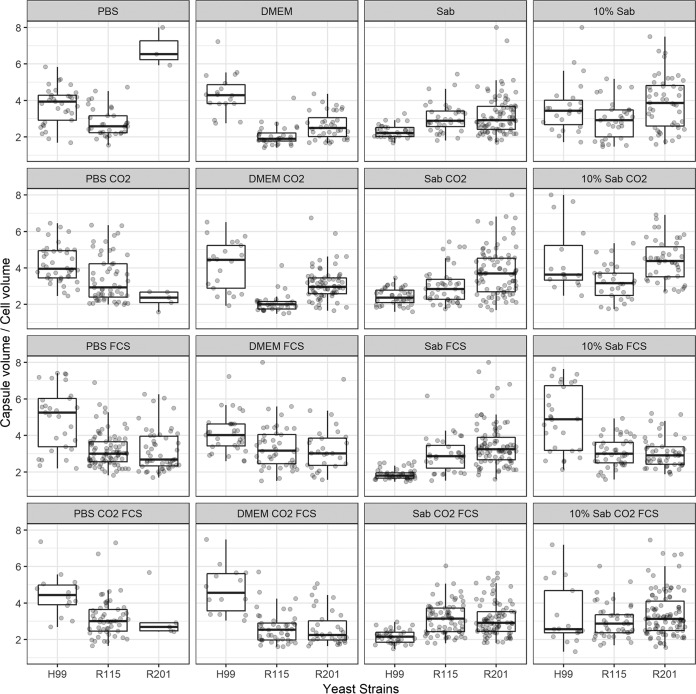
Clinical R. mucilaginosa strain 201 produces larger capsules than environmental R. mucilaginosa strain 115. The same experiments were performed as described for Fig. 3 for the following strains: H99 C. neoformans, ARY 115 environmental R. mucilaginosa, and ARY 201 clinical R. mucilaginosa. After incubation of equivalent numbers of cells in PBS, DMEM, Sabouraud, or 10% diluted Sabouraud media, with or without CO_2_ and with or without 10% FCS (as described in Materials and Methods and the Fig. 3 legend), samples were mixed with India ink. Four to eight pictures were taken for each of the 16 conditions for each of the three strains to capture random and representative images. The diameters of the cell and capsule and the cell alone without capsule were measured in ImageJ for as many cells as could be cleanly determined in each image. The ratios of cell volume with capsule (*V*_Tot_) and cell volume without capsule (*V*_cell_) are shown in gray dots. The bottom and top edges of the box plot correspond to the first and third quartiles of the data, respectively, with the vertical lines corresponding to no greater than 1.5 × the interquartile range. The middle line of each box represents the median of the data. Each condition was well represented by multiple measurements, with the exception of strain 201 in PBS, in which too few cells were noted with measureable contrast.

Capsule induction is often studied in C. neoformans by placing cells under starvation conditions and imaging the results with India ink; however, R. mucilaginosa strains have not been studied this way. We chose to observe cells in phosphate-buffered saline (PBS), Dulbecco’s modified Eagle's medium (DMEM), Sabouraud medium, and diluted (10%) Sabouraud broth under conditions maintained with and without the addition of fetal calf serum (FCS) and with and without the addition of CO_2_ (29, 30). For initial condition testing, we used C. neoformans H99 for comparisons with one clinical R. mucilaginosa strain (strain 201) and one environmental strain (strain 115) (Table 3; see also Fig. 3) (Fig. 4). In binning under each of the four medium conditions (regardless of the presence or absence of FCS or CO_2_), the R. mucilaginosa strains exhibited larger capsule ratios in Sabouraud media than the C. neoformans strains, but we observed the opposite result under each of the other three medium conditions (Table 3). None of tested conditions caused either R. mucilaginosa strain to produce capsules to the extent seen with strain H99 (Fig. 3). Under every set of conditions, the clinical 201 R. mucilaginosa strains produced larger capsules than the environmental 115 strain (Fig. 4). We noted some changes in cell size under some of these conditions, such as enlarged cells in DMEM for both tested R. mucilaginosa strains and even some elongated germ-tube-like structures for clinical strain 201 under DMEM- and Sabouraud-containing conditions. R. mucilaginosa cells often appeared to be more closely clumped together than C. neoformans, and this is quite visible in the India ink images (Fig. 3).

**TABLE 3:** R. mucilaginosa strains have larger capsule ratios than C. neoformans strains in Sabouraud media^*a*^

Medium	*V*_tot_/*V*_cell_			*P* value		
	H99 (*n*)	R115 (*n*)	R201 (*n*)	H99/R115	H99/R201	R115/R201
PBS	4.5 ± 1.6 (121)	3.2 ± 1.0 (221)	3.4 ± 1.7 (64)	<0.0001	<0.0001	0.3286
DMEM	5.1 ± 3.6 (92)	2.6 ± 1.0 (150)	3.0 ± 1.1 (168)	<0.0001	<0.0001	0.0025
Sab	2.2 ± 0.4 (154)	3.1 ± 0.9 (185)	3.4 ± 1.2 (339)	<0.0001	<0.0001	0.0003
TEN	6.7 ± 8.4 (92)	3.1 ± 0.9 (150)	3.7 ± 1.4 (168)	<0.0001	<0.0001	<0.0001

a Capsule induction was measured for H99 C. neoformans, ARY 115 environmental R. mucilaginosa, and ARY 201 clinical R. mucilaginosa under conditions of exposure to phosphate-buffered saline (PBS), Dulbecco’s modified Eagle’s medium (DMEM), Sabouraud (Sab) medium, or 10% diluted Sabouraud media (TEN). Data represent average capsule ratio ± standard deviation of total cell volume with capsule (*V*_tot_) to cell volume without capsule (*V*_cell_) of the different strains under the four different medium conditions tested (each medium condition was also examined with or without CO_2_ and with or without 10% FCS, but those data have been collapsed for this analysis). *n*, number of cells analyzed; *P* value, result from unpaired Welch’s two-sample *t* test as implemented in R. By this analysis, C. neoformans strain H99 had a larger capsule ratio value in PBS, DMEM, and 10% diluted Sabouraud media; however, R. mucilaginosa had a larger capsule ratio value in normal Sabouraud media.

### A *Rhodotorula*-specific anticapsular antibody was generated that bound all R. mucilaginosa strains.

**FIG 5:**
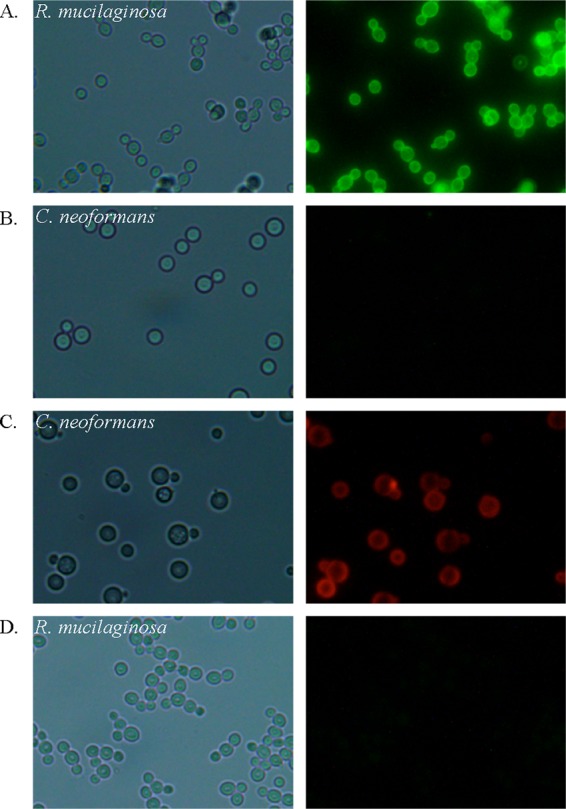
The anticapsular anti-*Rhodotorula* antibody is specific for *Rhodotorula* cells, and the *Cryptococcus* antibody is specific for *Cryptococcus*. (Left panels) bright-field images. (Right panels) Fluorescent images with appropriate cubes for each fluorophore. (A) R. mucilaginosa cells were incubated first with the purified anticapsular anti-*Rhodotorula* antibody (Rh1) and then with goat anti-rabbit IgG-FITC antibody. (B) C. neoformans cells incubated as described for panel A for R. mucilaginosa. (C) C. neoformans cells incubated with purified anticapsular anti-*Cryptococcus* antibody (2C3) directly conjugated with Cy3. (D) R. mucilaginosa cells incubated as described for panel C for C. neoformans. All images were generated at the same magnification and in the same way.

We wanted to study the capsule and cell surface of the R. mucilaginosa cells in manner similar to what has been done for C. neoformans, with fluorescent probes to the capsule, as described previously by Reese and Doering (31), and therefore pursued the production of rabbit antibodies to capsule material. We observed that the term bleed of rabbit 1 (of two) produced the most uniform and clear fluorescent rings around *Rhodotorula* cells and that antibody binding was specific to *Rhodotorula* species (Fig. 5A) and also observed that the antibody did not bind to cryptococcal cells (Fig. 5B). We have named this *Rhodotorula*-specific antibody Rh1. Furthermore, we observed that the *Cryptococcus* anticapsular antibody bound specifically to cryptococcal cells (Fig. 5C) and did not bind to rhodotorular cells (Fig. 5D).

### All tested *Rhodotorula* strains bound the Rh1 anti-capsule antibody, whereas C. neoformans strains did not.

Samples of each of the 16 R. mucilaginosa strains were incubated with the purified primary anti-*Rhodotorula* anti-capsule antibody (Rh1) followed by the goat anti-rabbit IgG fluorescein isothiocyanate (FITC) secondary antibody and visualized for the uniformity of fluorescent rings. All of the environmental and clinical strains picked up the antibody and produced intense and uniform ring fluorescence (see Table 4) similarly to the manner in which C. neoformans picks up its respective anti-capsule antibodies (31, 32). In fact, all of the *Rhodotorula* samples in our laboratory collection have been able to bind the Rh1 antibody, regardless of their genotyped species. We have tested nearly 50 strains.

**TABLE 4:** All 16 *R. mucilaginosa* strains bound in the anti-*Rhodotorula* capsule antibody assay, as well as in the WGA, CFW, and ConA assays, but did not bind EY tightly^*a*^

ARY strain	Intensity of fluorescence				
	Rh1	WGA	CFW	ConA	EY
*Rhodotorula*, environmental					
115	+++	+++	+++	+++	±
116	+++	+++	+++	++	−
120	+++	+++	+++	++	−
126	+++	+++	+++	++	±
162	+++	+++	+++	+++	−
200	+++	+++	+++	++	−
225	+++	+++	+++	+++	+
250	*+++*	+++	+++	+++	−

*Rhodotorula*, clinical					
201	+++	+++	+++	++	−
202	+++	+++	+++	+++	−
203	+++	+++	+++	++	−
204	+++	+++	+++	++	−
205	+++	+++	+++	++	±
206	+++	+++	+++	+++	±
207	+++	+++	+++	++	±
208	+++	+++	+++	++	±

*Cryptococcus,* clinical					
101 (H99)	−	++	++++	+++	++
177 (NIH 117)	−	++	++++	++	++

a R. mucilaginosa and C. neoformans strains were grown in YPD media overnight at 30°C, and equivalent cell amounts were incubated with the anti-*Rhodotorula* capsule antibody, Rh1, followed by the FITC-conjugated goat anti-rabbit IgG secondary antibodies as described in Materials and Methods and shown in Fig. 5. Surface probes of FITC-WGA, CFW, EY, or FITC-ConA were incubated as described in Materials and Methods and shown in Fig. 6. The intensity of fluorescence was scored, in a manner similar to what was reported previously for C. neoformans (42), as follows: ++++, intense fluorescence (generated for C. neoformans with CFW after viewing these cells); +++, bright fluorescence; ++, moderate fluorescence; +, low fluorescence; ±, thin rim of fluorescence (seen after viewing EY-tagged cells); −, no fluorescence. Cells were randomized before being viewed, and sample sets were viewed consistently by one individual.

### R. mucilaginosa cells bound WGA tightly, CFW slightly less tightly, and EY considerably less tightly than and ConA about the same as C. neoformans cells.

To further characterize the accessible surface, the 16 R. mucilaginosa strains and two cryptococcal control strains were each incubated with FITC-conjugated wheat germ agglutinin (WGA), calcofluor white (CFW), eosin Y dye (EY), and FITC-conjugated concanavalin A (ConA) separately and cells were then visualized with fluorescent imaging to observe surface-binding patterns.

Binding of WGA is interpreted to demonstrate exposed chitin and chitooligomers (24). All 16 R. mucilaginosa strains appeared to bind WGA more tightly than C. neoformans, as indicated by the presence of rings showing brighter fluorescence, suggesting that these components are more accessible in *Rhodotorula* strains, at least under these conditions (Table 4; see also Fig. 6). Calcofluor white (CFW) binds to both chitin and chitosan of the cell wall, and, because of its smaller molecular size, CFW is thought to indicate the total level of chitin and chitosan content (24). All of the R. mucilaginosa strains bound CFW with high intensity, but C. neoformans cell binding was even brighter. Our data show that R. mucilaginosa strains bound CFW at lower levels than C. neoformans (Table 4) (Fig. 6), suggesting that the total chitin and chitosan content in the *Rhodotorula* cells may have been less than in the C. neoformans cells. Eosin Y dye binds specifically to chitosan (17, 24). Cryptococcal cells bound the eosin Y more tightly and brightly than R. mucilaginosa, where the fluorescent rims were much thinner if even visible (Table 4) (Fig. 6), suggesting the level of chitosan is lower in the rhodotorular walls. This result also supports the idea of the reduction of CFW binding referred to above and may support the idea that the reduction of chitosan levels accounts for the difference.

**FIG 6:**
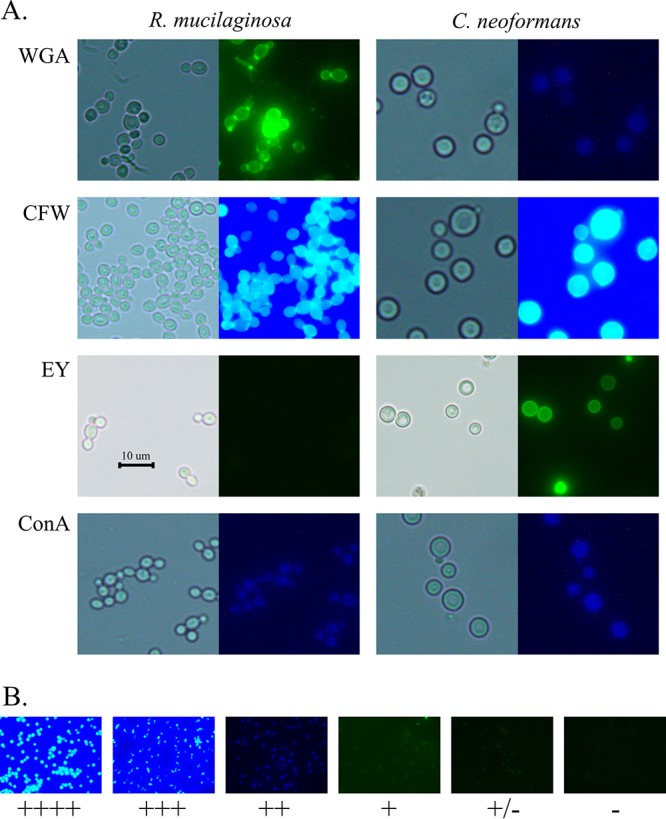
R. mucilaginosa cells bind WGA tightly, CFW slightly less tightly, and EY considerably less tightly than and ConA about the same as C. neoformans. (A) R. mucilaginosa ARY 115 and C. neoformans H99 cells were grown in YPD media overnight at 30°C and equivalent cell amounts incubated with FITC-WGA, CFW, EY, and FITC-ConA as described in Materials and Methods and Table 4. (Left panels) Bright-field images. (Right panels) Fluorescent images with appropriate cubes for the fluorophores used. (B) Example images of fluorescent intensity as scored in Table 4 are indicated as follows: ++++, intense fluorescence; +++, bright fluorescence; ++, moderate fluorescence; +, low fluorescence; ±, thin rim, image not easily captured; −, no fluorescence. Cells were generally randomized before viewing, and all sets were viewed by one person and generated in the same manner and at the same magnification. All images were generated at the same magnification and in the same way.

Concanavalin A (ConA) has been used to monitor mannose on fungal cell surfaces, such as on C. neoformans cells (18, 24). All of the R. mucilaginosa strains bound ConA with at least moderate intensity, and four environmental strains (ARY 115, 162, 225, and 250) and two clinical strains (ARY 202 and 206) bound ConA with high intensity. C. neoformans control strains bound ConA with moderate or high intensity. The binding of ConA suggests that the accessible mannoproteins of the R. mucilaginosa cells and C. neoformans cells are relatively similar under these conditions.

## DISCUSSION

We set out to characterize R. mucilaginosa strains collected from the environment and from patients and to compare them to C. neoformans strains in terms of their growth under cell envelope-challenging conditions, ability to undergo melanization, capsule production, susceptibility to common antifungals, and surface accessibility. We found that the strains obtained from various environmental locations or clinical specimens did not appear to behave uniformly phenotypically and biochemically, despite all being typed as R. mucilaginosa. They also behaved differently from strains of C. neoformans. These differences are summarized as follows.

Phenotypic and biochemical characterization of an organism provides a greater understanding of how the organism survives in different environments. Understanding the biogenesis and integrity of a cell wall and cell membrane could be useful in future antifungal production or applications. We hypothesized that the R. mucilaginosa strains would be susceptible to various conditions in a manner similar to that seen with C. neoformans, and we predicted that the clinical R. mucilaginosa strains would be more robust than the environmental ones. The R. mucilaginosa strains showed marked growth inhibition on YPD agar plates containing 1 mg/ml Congo red, whereas the cryptococcal strains were not affected. Furthermore, greater numbers of environmental strains were inhibited than clinical strains (two environmental strains exhibited at least some growth in comparison to five clinical strains; see Fig. 2). From this we conclude that there are differences between rhodotorular and cryptococcal cell wall polymer (beta-d-glucans or chitin) composition and/or assembly.

The literature demonstrates that wild-type cryptococcal strains (H99) grow well on media with 0.02% SDS (18), 0.05% SDS (33) and 0.06% SDS (24). We initially plated serial dilutions on YPD agar plates with 0.05% SDS, but we then dropped the concentration of SDS to 0.01% in order to view growth of R. mucilaginosa strains, and at that concentration, several strains grew at an order of magnitude less, suggesting that the membranes of R. mucilaginosa cells are not as tolerant of surfactant interruptions as cryptococcal strains.

Our data demonstrated that R. mucilaginosa strains were more robust in their growth on YPD plates with 1 mg/ml caffeine than what we observed for cryptococcal growth (the literature reports for cryptococcal cells on caffeine plates [24] support what we observed). The excellent growth of both environmental and clinical R. mucilaginosa strains in 1 mg/ml caffeine (Fig. 2) suggests potential signaling differences between R. mucilaginosa and C. neoformans.

The production of urease enzyme and the conversion of urea to ammonia and carbamate in C. neoformans have been demonstrated to enhance invasion into the central nervous system (CNS) (27). R. mucilaginosa associated with endocarditis has been demonstrated to be positive for urease production (34). Our urease data suggest that all of the strains hydrolyze urea, than there is an inherent range in terms of hydrolysis rates across the strains, and that clinical isolation does not enhance urease reaction capacity. Whether or not the production of urease in R. mucilaginosa strains allows the cells better access to the CNS is unclear, but urease could theoretically facilitate the activity of R. mucilaginosa in cases of meningitis.

Our India ink studies (Table 3) (Fig. 3 and 4) suggested that the capsule sizes of R. mucilaginosa are not nearly as expansive as those of C. neoformans, at least under the conditions employed. This is not totally surprising. Strains of *Rhodotorula* have been described as having a small capsule (11), and C. neoformans has traditionally been considered the only encapsulated pathogenic fungus. Our C. neoformans capsule results are in line with those seen previously (29, 30), and some of the variability in our measurements of total capsule plus cell volume may have been due to the length of time of our incubation, as discussed previously by Zaragoza et al. (29). Under every set of conditions, the clinical strain 201 capsules were larger than the environmental R. mucilaginosa strain 115 capsules. While not necessarily correlated to strain 201 having been originally isolated from a patient, it is interesting that the clinical strain produced larger capsules. Intriguingly, capsule ratios in Sabouraud media were larger for R. mucilaginosa strains (i.e., for both representative clinical strain 201 and environmental strain 115) than for the C. neoformans strain, underscoring the differences in the yeast cells and the importance of testing various conditions.

The smaller capsules of R. mucilaginosa could contribute to greater accessibility of chitin and chitosan, which would fit with our data showing WGA binding more abundantly and brightly to R. mucilaginosa strains than to C. neoformans (Table 4). Because of its smaller molecular size, CFW is thought to indicate the total content level of chitin or chitooligomers that could be reached by the dye (24). Our data show R. mucilaginosa strains binding CFW less than was bound by C. neoformans (Table 4), suggesting that the total content of chitin or chitooligomers in R. mucilaginosa cells may be less than in C. neoformans cells. This is further supported by our EY binding data (Table 4) demonstrating low binding levels and low levels of chitosan in R. mucilaginosa cells. In conclusion, we take these findings to suggest that the amounts of chitin and, particularly, of chitosan are lower in R. mucilaginosa cells but that these components are more accessible than in C. neoformans.

All of the R. mucilaginosa strains were resistant to fluconazole. Only two of the clinical strains (ARY 202 and 204) were susceptible to voriconazole, while all of the environmental R. mucilaginosa strains were resistant to voriconazole (Table 2). In contrast, all four of the tested C. neoformans strains were susceptible to voriconazole, and all four were either susceptible or susceptible in a dose-dependent manner (1) to fluconazole. These results are in line with what has been published previously for the same method, where the majority of isolates of R. mucilaginosa strains were resistant to fluconazole, there was a range of responses to voriconazole, and C. neoformans strains were generally susceptible to voriconazole and fluconazole (1). We also note that just because our environmental strains were isolated from environmental sites does not mean that they could not cause clinical infections. But our data suggest that if they did, they would not respond to these antifungal agents. We did not see any specific correlation between the two voriconazole-susceptible clinical strains and their growth on cell wall integrity plates that we could link with the antifungal results.

Our capsule analysis studies performed using an antibody to the R. mucilaginosa capsule demonstrated the potential value of the use of such a tag to identify and study putative *Rhodotorula* cells collected from both clinical and environmental settings (Table 4) (Fig. 5). Currently, a major clinical challenge in treating infections caused by *Rhodotorula* spp. is that of not expecting its presence. Given that *Rhodotorula* strains do not respond to the first-line antifungal caspofungin, this delay in identification can have serious consequences. Susceptibility tests have demonstrated that *Rhodotorula* species respond best to amphotericin B and flucytosine and very poorly to the azoles (1, 7–10). Our data corroborate this lack of efficacy of azoles for control of environmental strains as well. Availability of a specific antibody could be useful in rapid diagnostic approaches for detection of infections caused by *Rhodotorula* spp. To our knowledge, this is the first work that has studied the capsule of R. mucilaginosa with any anti-capsule antibodies or with a focus on the capsule of this fungal pathogen.

Taken together, the cell envelope integrity, capsule analysis, and fluorescent-probe binding results presented here suggest that the pathways and composition of R. mucilaginosa cell walls and capsules are different from those of cryptococcal cells. R. mucilaginosa appears to have less chitin and chitosan, though these cell wall components may be more accessible than in C. neoformans, perhaps because of the presence of thinner capsules. The marked reduction of R. mucilaginosa levels on Congo red media may suggest an impact on the production or assembly of glucan or chitin levels in these cells. The robust growth of R. mucilaginosa on caffeine-containing plates supports the idea that the signaling pathways of these cells differ from those of C. neoformans regarding cell wall stability and repair. The R. mucilaginosa strains from clinical sources exhibited a slightly higher tolerance of cell wall integrity challenges and larger capsules than were shown by strains from environmental sources, and the environmental strains exhibited higher levels of resistance to antifungal agents. This work suggested that future studies that dig deeper into the cell wall and capsule composition of *Rhodotorula* spp. could prove fruitful and might inform the efficacy of future antifungal treatments or antifungal production. Our generation of the Rh1 antibody specific to *Rhodotorula* fungi could be a useful diagnostic tool, and the present report is the first known report of such a tool in the literature.

## MATERIALS AND METHODS

### Strains and strain maintenance.

Eight clinical R. mucilaginosa strains isolated from blood were obtained from the Medical Microbiology Division at the University of Iowa Carver College of Medicine, Iowa City, IA, as listed in Table 1. Environmental *Rhodotorula* strains were collected by various members of the Reese laboratory from locations in St. Louis, MO, or from locations in Allentown, PA, or from other locations in Pennsylvania or were purchased as a control strain from Wards or Carolina (Table 1). Clinical Cryptococcus neoformans strains (var. *grubii* serotype A H99 and var. *neoformans* serotype D JEC21) were obtained from Tamara L. Doering (Washington University School of Medicine, St. Louis, MO) and the NIH strains from Arturo Casadevall (Johns Hopkins University). Strains were stored at −80°C in yeast extract-peptone-dextrose (YPD) media with 15% glycerol until utilized and were cultured on YPD agar plates at 30°C for study.

### Isolation of genomic DNA.

Genomic DNA (gDNA) was extracted using a Zymo fungal/bacterial DNA extraction kit (Zymo catalog no. D6005), and approximately 100 mg of each strain was scraped from growth on a YPD agar petri dish. Kit protocols were followed, save for the bead beater time being reduced to two separate 2-min intervals, for a total of 4 min per sample, to avoid overheating of tubes. Yields of gDNA ranged from 5 ng/ml to 30 ng/ml as determined by the use of NanoDrop ND-1000 spectrophotometer (NanoDrop) readings. Alternatively, for some strains, gDNA was extracted from overnight YPD liquid cultures with a PowerSoil DNA isolation kit (Mo Bio Laboratories), according to the manufacturer’s protocol, and was quantified by Qubit 3 Fluorometer (Invitrogen) readings.

### Amplification of internal transcribed spacer (ITS) regions.

ITS regions were amplified using standard fungal methods (35) with either Qiagen *Taq* PCR master mix (Qiagen, catalog no. 201443) or REDTaq ReadyMix PCR mix (Sigma-Aldrich Inc., catalog no. R2523), and primers ITS1 (5′TCCGTAGGTGAACCTGCGG-3′) and ITS4 (5′-TCCTCCGCTTATTGATATGC-3′) (36). Amplification conditions employed were as follows: 94°C (3 to 4 min); 30 to 35 cycles of 94°C (1 min), 55°C (2 min), 72°C (1 to 2 min), and 72°C (7 min); a hold at 4°C. Amplicons were confirmed using DNA gel electrophoresis with 1% agarose gels in 1× Tris-acetate-EDTA (TAE) or 1× Tris-borate-EDTA (TBE) buffer and visualized with ethidium bromide, with the expected product being about 500 bp in size.

### ITS region sequencing and analysis.

Amplicons were cloned into a TOPO TA cloning kit for sequencing (Invitrogen), and duplicate Sanger sequencing reactions were performed with M13 primers at the Protein and Nucleic Acid Chemistry Laboratory at the Washington University School of Medicine. Trace data were analyzed and assembled into contigs, and all of the strains in the full set currently under study in the laboratory (about 50 strains) were genotyped via targeted-locus nucleotide BLAST (Basic Local Alignment Search Tool). Only strains that matched R. mucilaginosa were selected for further comparative analysis (see Table 1) (Fig. 1).

Molecular phylogenetic analysis was performed by the maximum likelihood method. The evolutionary history was inferred by using the maximum likelihood method based on the Kimura 2-parameter model (37). The initial tree(s) for the heuristic search was obtained automatically by applying Neighbor-Join and BioNJ algorithms to a matrix of pairwise distances estimated using the maximum composite likelihood (MCL) approach and then selecting the topology with the superior log likelihood value. Evolutionary analyses were conducted in MEGA7 (38). Multiple isolates of pink yeast were sequenced and analyzed, and only the strains whose best match was R. mucilaginosa and which formed a consistent clade with known R. mucilaginosa strains were used for the studies presented here.

### Cell wall integrity analysis.

In tests of cell wall integrity, exposure of organisms such as Cryptococcus neoformans and Saccharomyces cerevisiae to different conditions has been reported in the literature and we employed some of these here to assess the cell envelopes of our strains, including Congo red, calcofluor white, sodium dodecyl sulfate, caffeine, hydrogen peroxide, and sodium chloride (16–18, 25). Strains were grown in liquid YPD culture overnight, diluted to an optical density at 600 nm (OD_600_) of 0.1, serially diluted 5-fold in YPD liquid media, and spotted in 5-μl increments on two plates of YPD medium alone or YPD medium containing 0.05% SDS (Bio Rad Laboratory catalog no. 161-0301) or 0.5 or 1.0 mg/ml calcofluor white (Fluorescent Brightener 28; Sigma catalog no. F-3543) prepared from a stock solution at pH 11; 1.5 mg/ml caffeine (Sigma catalog no. C-0750); 0.5% Congo red (Fisher Laboratory Chemical catalog no. A-795) prepared from a stock solution with 50% ethanol; 1.2 M NaCl (Research Products International Corp. catalog no. S23020); or 1 mM hydrogen peroxide (Walgreens) (3%). Two replicate plates corresponding to each condition were incubated at 25°C and two at 37°C for 4 days before the plates were scanned with an Epson color scanner, images were captured with VueScan ×64 software, and Photoshop CS4 was used to prepare figures.

### Urease production assay.

Three separate 2-ml aliquots of urease media (Remel) were inoculated with 2.5 × 10^7^ cells of each strain and incubated at 37°C for 7 days. Samples were viewed on a nearly daily basis to determine which samples hydrolyzed urea more quickly than others and were categorized for any change in the color of the medium, with a change from light pink to hot pink indicating a positive reaction for the hydrolysis of urea. All 16 R. mucilaginosa strains were tested and compared to C. neoformans strains H99 and NIH 117.

### Melanin production assay.

Strains were grown in liquid YPD culture overnight, diluted to an OD_600_ of 0.1, serially diluted 5-fold in YPD liquid media, and spotted in 5-μl increments onto plates of minimal medium (10 mM MgSO_4_, 29.4 mM KH_2_PO_4_, 13 mM glycine, and 3 μM thiamine, pH 5.5) supplemented with 1.5 mM glucose and 1 mM l-DOPA, modified from the composition previously reported by Eisenman et al. (39) to have more dextrose.

### Antifungal susceptibility.

Strains were grown in liquid YPD culture overnight and diluted to an OD_600_ of 0.1, 1 ml was spread across 150-mm-diameter Mueller-Hinton plates (Remel lot 28991) with a sterile swab, and the plates were rotated 90° and spread further and finally rotated 90° and swabbed again for thorough spreading. Sterile tweezers were used to apply two antifungal disks of fluconazole and two of voriconazole to each sample. Plates were incubated at 37°C for 2 days, scanned, and measured.

### India ink capsule visualization.

Strains were grown in liquid YPD culture overnight, and the concentrations were determined at OD_600_ via a spectrophotometer (OD_600_ of 1 = 1 × 10^7^ cells). Aliquots of 5 × 10^6^ cells per well were washed in the appropriate growth condition (all samples maintained under the same conditions were washed in bulk) and resuspended in the same growth media and added to the wells in 100-μl volumes. In cases in which fetal calf serum was a part of the condition, the fetal bovine serum (Sigma F2442) was added in 200-μl aliquots to reach 10% of the final concentration. Medium conditions included phosphate-buffered saline (PBS), DMEM (with 4.5 g/liter d-glucose and l-glutamine and 110 mg/liter sodium pyruvate [Gibco 11995-065]), Sabouraud (BD 238230), and Sabouraud diluted to 10% to reach a final volume of 2 ml per well. Plates for CO_2_ conditions were placed in a plastic bag all at one time, CO_2_ was added to the bag, and the bag and contents were incubated at 37°C for 7 days. A dot of India ink (Hardy Diagnostics Z64) and a loopful of the well samples were transferred to slides and mixed for visualizing capsules. The cells were viewed with a Zeiss Scope A.1 microscope at ×630, images were captured with an AmScope 5.2-megapixel (MP) camera, and Photoshop CS4 was used to prepare figures.

For each of the three strains and 16 conditions, four images were captured in a general diagonal pattern across the slide, with photographing of up to four additional images around the slide performed if insufficient cells were observed in the original set of four samples. Images were batch processed in the Fiji distribution of ImageJ (40) in order to standardize measurement of the cell diameters. Each image was converted to 8-bit type, and the threshold value was set to 221 to create a black India ink background with distinct capsule borders. For each cell, the diameter of the cell capsule and the diameter of the cell membrane were measured in ImageJ and converted to a volume value using the equation 4/3 × π × (diameter/2)^3^. The ratio reported is the total cell volume (*V*_Tot_) divided by the cell volume without capsule (*V*_cell_). These methods are comparable to those used to calculate capsule volumes for C. neoformans in the literature (29, 30).

### Preparation of capsule material for antibody production.

R. mucilaginosa strain ARY 115 conditioned media were generated by culturing cells inoculated from a freshly growing plate into 25 ml minimal media (BD Difco yeast nitrogen base without amino acids; catalog no. DF0919) for 4 to 5 days at 30°C with shaking (New Brunswick Scientific Co.; Innova 4000 table top shaker) at around 225 rpm. This strain was selected as it was the original *Rhodotorula* strain in the Reese laboratory and had been treated as a reference strain. The cells were pelleted at low speed (Beckman Coulter Allegra 25R centrifuge), and the supernatant was subjected to sterile filtration and buffer exchange into phosphate-buffered saline (PBS; pH 7.4) (Centricon centrifugal filter device kit by Millipore) in order to obtain the shed capsule material without extraneous medium components. A phenol sulfuric acid assay was used to determine the concentration of carbohydrate in the sample (method adapted from reference 41).

### Generation of rabbit sera against *Rhodotorula* capsule.

Samples of capsule determined to contain 0.2 mg carbohydrate were sent to Lampire Biological Laboratories (Pipersville, PA) for inoculation into two separate rabbits. Serum samples were obtained from each rabbit for control experiments and possible antibody synthesis at the following time points: before the injection, 30 days postinjection, 50 days postinjection, and at the final termination bleeds.

### Testing of rabbit antisera for binding to *Rhodotorula* capsule.

Overnight cultures of both R. mucilaginosa strain ARY 115 and C. neoformans strains JEC21 and H99 were prepared. The cells were counted with a hemocytometer or measured by spectrophotometer, and aliquots of 2.5 × 10^6^ cells/μl were centrifuged for 1 min at high speed on a tabletop minicentrifuge (e.g., at 14,000 rpm with the Eppendorf 5415D Microcentrifuge) and were washed once with 1 ml of PBS for each of the testing conditions. Samples were resuspended in 250 μl of 1% bovine serum albumin (BSA)–1% PBS (to decrease nonspecific binding), and 25 μl of each antiserum to be tested (or PBS to control for the absence of antibody) was added and incubated at room temperature with rotation for 30 min as the primary antibody incubation step. Cells were centrifuged and washed in 1 ml of PBS to remove excess primary antibody. Cells were resuspended in 49 to 250 μl of 1% BSA–1% PBS and incubated with anti-rabbit IgG conjugated to fluorescein isothiocyanate (FITC) from goat (Alfa Aesar BT 557; catalog no. J64967) as a secondary antibody to reach final concentrations of 1 to 10 μg/ml and rotated at room temperature for 30 min. C. neoformans controls using anticapsular antibody directly conjugated with a fluorophore, such as 3C2-Cy3, were also used to serve as fluorescence controls (requiring alternative filters) as previously described (31). Cells were viewed using fluorescence microscopy.

### Purification of the *Rhodotorula* antibody and strain analysis.

The terminal bleed rabbit sera from rabbit 1 were purified using a protein A IgG purification kit (Thermo Scientific). The eight clinical and eight environmental strains were incubated with the primary and secondary antibodies as described above and assessed for binding of the anti-*Rhodotorula* anticapsular antibody.

### Immunofluorescence visualizing and imaging.

The cells were viewed with a Zeiss Scope A.1 microscope at ×630, images were captured with an AmScope 5.2-MP camera using appropriate excitation cubes, and Photoshop CS4 was used to prepare figures. If an uninterrupted and uniform fluorescent ring was visible surrounding the cell, this was interpreted as indicating that the primary and secondary antibodies attached to the cell were present as described previously (32).

### Surface characterization of *Rhodotorula* cells.

The 16 R. mucilaginosa strains were screened for their ability to bind the primary antibody (Rh1) and secondary antibodies for the capsule (as described above). To probe the accessible surface of the different strains (in a manner similar to that performed with C. neoformans strains as described by Reese and Doering [31]), aliquots of 2.5 × 10^6^ cells/μl to 2.5 × 10^7^ cells/μl grown in YPD media were incubated with FITC-wheat germ agglutinin (Invitrogen catalog no. W834) at final concentrations of 0.1 to 0.5 mg/ml in 1% BSA or with FITC-concanavalin A (Molecular Probes) at final concentrations of 0.1 to 0.4 mg/ml in 1% BSA for 60 min at room temperature. Samples tagged with eosin Y dye (final concentration of 250 μg/ml) were washed and resuspended in McIvaine’s buffer (0.2 M Na_2_HPO_4_, 0.1 M citric acid, pH 6.0) and incubated for 15 min at room temperature. Cell aliquots were also incubated with up to 1 ampule of calcofluor white (BD BBL diagnostic stain reagent; catalog no. 261195) for 30 min with rotating at room temperature. In each case, samples were incubated and washed in PBS before viewing. Samples were generally randomized before assessment for fluorescence visualization as described above, and all samples tagged with the same probe were viewed by the same individual.
